# Effectiveness of an Interactive Workshop in Increasing Awareness About Oral Cancer Screening Among Dental Undergraduate Students: A Pre- and Post-experimental Study

**DOI:** 10.7759/cureus.84723

**Published:** 2025-05-24

**Authors:** Uma V Datar, Mamata Kamat, Mahesh R Khairnar, Umesh Wadgave, Karishma Desai

**Affiliations:** 1 Oral Pathology and Microbiology, Bharati Vidyapeeth (Deemed to be University) Dental College and Hospital, Sangli, Sangli, IND; 2 Public Health Dentistry, Faculty of Dental Sciences, Institute of Medical Sciences, Banaras Hindu University, Varanasi, IND; 3 Public Health Dentistry, ESIC (Employee's State Insurance Corporation) Dental College, Kalaburagi, IND; 4 Oral and Maxillofacial Pathology, Bharati Vidyapeeth (Deemed to be University) Dental College and Hospital, Navi Mumbai, Navi Mumbai, IND

**Keywords:** dental education, early oral cancer detection, oral cancer, oral cancer screening, oral potentially malignant disorders (opmd), toluidine blue staining

## Abstract

Background

Oral cancer (OC) screening plays a pivotal role in the early detection of OCs, which helps improve the patient's prognosis. Hence, training students is essential to facilitate the opportunistic screening of patients. With this objective, we arranged an interactive workshop and assessed the program's effectiveness.

Methods

This experimental study was conducted on 67 interns in a dental college, employing a pre-post experimental design. The participants were asked to answer a questionnaire before and after the workshop. This self-administered questionnaire, consisting of 45 questions, tested the students’ knowledge, attitude, and skills regarding OC screening. The workshop was not just about lectures - it was a hands-on experience. Interactive lectures were conducted on the epidemiology, causative and risk factors, clinical presentation, and early detection methods of OC and potentially malignant disorders (PMDs). A practical demonstration of toluidine blue was also a vital part of the workshop.

Results

Awareness lectures and hands-on training significantly improved knowledge (t-value = -19.681, p = 0.001) and attitude (t-value = -5.629, p = 0.001) before and after the workshop.

Conclusion

Periodic training of dental students for OC screening enables better clinical transition and promotes early diagnoses of OC and PMDs.

## Introduction

Global statistics of oral cancer (OC) in 2020 reported that 248,360 out of 377,713 cases of OC were from Southeast Asia. India accounted for 135,929 new cases annually, i.e., over 30% of the global cancer burden [1]. On one hand, the incidence of OC is on the rise, while the five-year survival rate of OC has not improved over the past few years [2]. Early diagnosis is the key to improving treatment outcomes. Oral potentially malignant disorders (OPMDs) are forerunners of OC. Without a national oral screening program, opportunistic screening by dentists is essential for early diagnosis and improved treatment outcomes. Several studies have assessed dental students’ knowledge, attitudes, and practices regarding cancer screening [3-11]. However, the literature regarding the usefulness of the most frequently used method to teach dental undergraduates OC screening methods is lacking. This article investigates the effectiveness of hands-on practice and interactive classes in teaching OC screening methods to dental undergraduates.

## Materials and methods

This experimental study, employing a pre-post design, was conducted among dental college interns. Prior approval was also obtained from the institutional ethics committee. A self-administered questionnaire (Appendices) was developed, comprising 45 questions to assess students’ knowledge, attitudes, and skills regarding OC screening. A pilot survey was conducted by self-administering the questionnaire to five faculty members to check the validity of the questionnaire using the Content Validity Index (CVI). The overall CVI of 0.83 is considered acceptable. The questionnaire was then administered to 10 participants (who were not part of the main study) to assess its internal consistency. The value of Cronbach’s alpha coefficient (internal consistency reliability) was 0.74, which is acceptable. The questionnaire was administered again to the same 10 participants after 10 days to determine test-retest reliability. The kappa coefficient value obtained was 0.746, which is considered good. The authors attempted to minimise response bias by avoiding leading questions in the questionnaire, not recording any personally identifiable data, and requesting that participants refrain from discussing the questionnaire with others while completing it.

All the interns were invited to participate in this research. Written informed consent was obtained from all the participants. A one-day OC screening workshop was planned, and 67 interns participated. The Public Health Dentistry, Oral Pathology, and Oral Medicine faculty delivered the lectures. Before the workshop began, the students were asked to complete the self-administered questionnaire. Interactive lectures, including didactic and hands-on sessions, were conducted on the epidemiology, causative and risk factors, clinical presentation, and early detection methods of OC and OPMD. We also conducted a practical demonstration session on using toluidine blue for screening purposes. Toluidine blue was selected because it is cost-effective for screening OC, with a sensitivity and specificity of 71% and 90%, respectively [12]. After that, the participants were asked to answer the same questionnaire following the workshop. The completed questionnaires were collected, and the data from the response sheets were entered into Microsoft Excel (Microsoft® Corp., Redmond, WA, USA). Statistical analysis was performed with IBM SPSS Statistics for Windows, Version 20 (released 2011; IBM Corp., Armonk, NY, USA), and descriptive statistics were employed. A paired t-test was conducted to compare the continuous data before and after the study. The Chi-square test was used to compare categorical data. Statistical significance was fixed at p ≤ 0.05.

## Results

Knowledge, attitude, and practice about cancer screening

Table 1 depicts the questions accurately answered by the participants before and after the workshop. Most participants’ knowledge regarding the etiology of OC was sound. However, only nine participants stated that they had adequate knowledge regarding the detection of OSCC, and only one felt that their knowledge was up to date before the session. In contrast, after the session, this number increased to 39 (p < 0.05). Similarly, before the session, none of the participants were confident in clinically diagnosing OC, but after the session, the number increased to four (p < 0.05). Only nine participants were confident in diagnosing nodal involvement before the workshop; this number increased significantly to 29 after the workshop (p < 0.05). Most students identified vital staining, cytology, and brush biopsy as cancer screening tests; however, a minority identified chemiluminescence (12 participants), Velscope (three participants), and colposcopy as adjuvant tests.

**Table 1:** Knowledge, attitude, and practice of cancer screening among the participants before and after the training Chi-square test; * indicates a significant difference at p ≤ 0.05.

Question		Number of participants who answered correctly		p-value
		Pre-test, n (%)	Post-test, N (%)	
Do you screen the oral mucosa of patients if they are in high-risk categories		59 (88.05)	64 (95.52)	0.207
Do you ask the patient's history about tobacco and alcohol use		67 (100)	67 (100)	1.000
Most dentists are adequately trained to examine patients for oral cancer		55 (82.08)	62 (92.53)	0.117
Do you feel you have adequate knowledge concerning about detecting of oral squamous cell carcinoma (OSCC)?		9 (13.43)	61 (91.04)	0.001*
My knowledge about oral cancer is up to date		1 (0.01)	39 (58.2)	0.001*
I am confident about diagnosing oral carcinoma clinically		0 (0)	4 (5.97)	0.119
I am confident about diagnosing nodal metastasis		6 (8.95)	29 (43.28)	0.001*
Which of the following is the most common type of oral cancer?		57 (85.07)	65 (97.01)	0.030*
Identified the etiological agents of OSCC	Tobacco use	64 (95.52)	67 (100)	0.244
	Alcohol consumption	48 (71.64)	65 (97.01)	0.001*
	UV light exposure	53 (79.10)	60 (89.55)	0.153
	Human papillomavirus 16 & 18	50 (74.62)	66 (98.5)	0.001*
	Occupation	46 (68.65)	53 (79.1)	0.238
	Genetic	44 (65.67)	62 (92.53)	0.001*
	Chronic trauma from a sharp tooth	54 (80.59)	63 (94.02)	0.035*
	Ill-fitting dentures	47 (70.14)	62 (92.53)	0.002*
	Poor dental hygiene	35 (52.23)	62 (92.53)	0.001*
Annual oral cancer examinations should be provided for those of 40 years of age and above		47 (70.14)	59 (88.05)	0.018*
Identified the precursors of OSCC	Oral submucous fibrosis	40 (59.7)	67 (100)	0.001*
	Leukoplakia	39 (52.20)	65 (97.01)	0.001*
	Erythroplakia	31 (46.26)	65 (97.01)	0.001*
	Lichen planus	37 (55.22)	61 (91.04)	0.001*
	Hairy leukoplakia	25 (37.31)	52 (77.61)	0.001*
	Smokers palate	22 (32.83)	53 (79.10)	0.001*
	Discoid lupus erythematosus	27 (40.29)	47 (70.14)	0.001*
	Xeroderma pigmentosum	5 (7.46)	31 (46.36)	0.001*
	Proliferative verrucous leukoplakia	49 (73.13)	63 (94.02)	0.002*
Does early detection of OSCC improve the survival status of OSCC		60 (89.55)	65 (97.01)	0.165
Are all the OSCCs preceded by potentially malignant disorders (PMDs)?		12 (17.91)	17 (25.37)	0.402
What is the commonest cause of oral submucous fibrosis?		58 (86.56)	61 (91.04)	0.585
Which of the following PMDs has the highest malignant transformation rate?		38 (56.71)	56 (83.58)	0.001*
Do patients without habits develop OSCC?		50 (74.62)	60 (89.55)	0.041*
Common site for OSCC in India		59 (88.05)	67 (100)	0.006*
Does TNM staging affect the therapeutic modality		58 (86.56)	66 (98.5)	0.017*
Biopsy should be performed on a non-healing ulcer on the tongue?		27 (40.29)	10 (14.92)	0.002*
Identified the clinical diagnostic aids of OSCC	Toludine blue (vital staining)	58 (86.56)	61 (91.04)	0.585
	Cytology	49 (73.13)	60 (89.55)	0.025*
	Brush biopsy	39 (52.20)	60 (89.55)	0.001*
	Chemiluminescence	12 (17.92)	39 (58.2)	0.001*
	Velscope	3 (4.47)	53 (79.1)	0.001*
	Colposcopy	1 (0.01)	28 (41.79)	0.001*
The test that establishes the final diagnosis of OSCC		43 (64.17)	58 (86.56)	0.001*
I am comfortable referring suspicious oral lesions to the specialists		64 (95.52)	67 (100)	0.244

Comparison of knowledge, attitude, and practice of cancer screening (before and after the workshop)

There was a significant improvement in each subdomain of knowledge regarding etiology (t-value = -9.177, p < 0.001), clinical diagnosis of OPMDs (t-value = -10.827, p = 0.001), and OCs (t-value = -16.740, p < 0.001) (Table 2).

**Table 2:** Comparison of knowledge subdomains (%) before and after oral cancer (OC) screening workshop Paired t-test; * indicates a significant difference at p ≤ 0.05. OSCC, oral squamous cell carcinoma; PMDs, potentially malignant disorders

Domain	Pre-test	Post-test	t-value	p-value
Etiology of OSCC	73.29 ± 18.29	92.54 ± 13.18	-9.177	0.001*
PMDs	50.34 ± 23.86	84.26 ± 16.49	-10.827	0.001*
Detection of OSCC	45.44 ± 17.05	80.43 ± 16.42	-16.740	0.001*
General about OSCC	55.65 ± 17.25	68.87 ± 10.82	-5.512	0.001*

On comparing the overall score (t-value = -19.619, p = 0.001), knowledge (t-value = -19.681, p = 0.001), and attitude (t-value = -5.629, p = 0.001) before and after the workshop, significant improvement was noted in all domains (Table 3).

**Table 3:** Comparison of knowledge and attitude (%) before and after oral cancer (OC) screening workshop Paired t-test; * indicates a significant difference at p ≤ 0.05.

Domain	Pre-test	Post-test	t-value	p-value
Overall	57.20 ± 11.49	81.26 ± 8.89	-19.619	0.001*
Knowledge	56.35 ± 12.99	82.65 ± 9.64	-19.681	0.001*
Attitude	94.03 ± 16.34	97.01 ± 14.77	-5.629	0.001*

A slight improvement in the students' practice domain was observed. Though relevant, this change was not statistically significant (Table 4).

**Table 4:** Comparison of practice before and after oral cancer (OC) screening workshop Chi-square test; * indicates a significant difference at p ≤ 0.05.

Practice	Response	Pre (%)	Post (%)	p-value
Do you screen the oral mucosa of high-risk patients?	Agree	88.0	95.5	0.103
	Not sure	6.0	3.0	
	Disagree	6.0	1.5	
Do you ask the patient about the history of tobacco and alcohol use?	Agree	98.5	100	1.000
	Disagree	1.5	0	

## Discussion

The incidence of OCs is expected to increase globally to approximately 28,887,940 (~50% of the present incidence), and in India, to 209,169 [1]. One way of averting this imminent crisis is through early detection or by identifying lesions in the premalignant stage through OC screening [13-17]. Though the Government of India envisages free cancer screening for all, the 1.35% GDP allocation to healthcare undermines this possibility [2]. In this context, opportunistic screening in dental colleges is vital to reducing the cancer burden. Dentists play a crucial role in the early diagnosis of OCs and OPMDs [4,17-19]. Training dental students to become proficient in OC screening is essential.

Knowledge is crucial in OC screening, as dental students must be informed and updated about the risk factors, symptoms, and diagnostic methods. In the present study, all the participants agreed that asking for tobacco history and screening high-risk patients is essential. Similar to our results, Gunjal et al. [4] and Srivastav et al. [20] reported that more than 90% and 75% of participants, respectively, could identify tobacco as an etiologic factor. Human papillomavirus (HPV) as an etiological factor was identified by 50% of participants, as reported by Keser and Pekiner [9]; however, our results were similar to those of Tarakji [21], i.e., 74%.

Surprisingly, 59% accurately identified OSMF as an OPMD, 52.3% identified leukoplakia, and only 46% identified erythroplakia. Carter and Ogden [11] reported that approximately 80% of dental students could recognize leukoplakia and erythroplakia as early changes in OC. Similarly, Sitheeque et al. [22] reported that 71% of dental students could identify erythroplakia as an OPMD. Periodic sessions on OC screening will inculcate positive changes in students pursuing private practice.

Improvement in attitude post-test

There was some disparity among the students regarding whether a biopsy should be performed on a non-healing ulcer. Interventions to promote education and desirable attitudes toward OC screening are needed.

Dental students must be trained to use proper clinical techniques for screening, such as visual examination, palpation, and the application of diagnostic/screening aids. Patient communication - an explanation of the need for a screening process and the importance of early detection - must be effectively demonstrated. Studies have elucidated that training and continuing education programs enhance the promptness of screening OCs, and other primary prevention activities are most effective in improving treatment outcomes [19]. Implementing screening programs by dental colleges nationwide will aid in early detection, improve survival rates, and decrease the economic burden of OC. This research was conducted on a limited sample from a single institution, so our findings may not directly apply to students in India or globally. However, this research highlights the potential for evaluating the effectiveness of an OC screening training program for dental interns, with a specific focus on operational strategies in the Indian subcontinent. It is important to note that further research is needed to determine the most effective methods for increasing awareness among students about OC screening. Additionally, long-term follow-up interventions are required to understand the impact of such training. To the best of our knowledge, this is the only experimental study with a pre- and post-study model. It was conducted only in a single dental college with 67 dental interns to measure immediate outcomes. 

## Conclusions

This trial demonstrated that awareness classes and hands-on workshops provided encouraging improvements in the knowledge and attitude of dental students regarding OC screening. There is a need to explore various methods and curricula to enhance the effectiveness of training in OC screening. Regular refresher courses, continuing dental education at the institutional level, and updated curricula by governing bodies are essential for translating the knowledge into practice.

## Appendices

Questionnaire on awareness of oral cancer screening

Thank you for your participation in this survey! This self-administered questionnaire is designed to assess the knowledge, attitude, and skills of dental interns in screening oral cancer cases. The information you provide will be used solely for our research. Your identity will not be disclosed. Please select a single appropriate option for each question and mark it with a “√”.

1. Age: __________

2. Gender: ________

3. Do you screen the oral mucosa of patients if they are in high-risk categories

a. Agree

b. Disagree

c. Not sure

4. Do you ask patient history about tobacco and alcohol use

a. Agree

b. Disagreey

5. Most dentists are adequately trained to examine patients for oral cancer

a. Agree

b. Disagree

c. Don’t know

6. Do you feel you have adequate knowledge concerning about detecting of oral squamous cell carcinoma (OSCC)?

a. Agree

b. Disagree

c. Don’t know

7. My knowledge about oral cancer is up-to-date

a. Agree

b. Disagree

c. Don’t Know

8. About diagnosing OSCC from clinical appearance you feel

a. Very confident

b. Confident

c. Unsure

d. Absolutely unsure

9. I am confident about cervical lymph node palpation to check for nodal metastasis

a. Agree

b. Disagree

c. Not sure

10. Which of the following is the most common type of oral cancer?

a. Oral squamous cell carcinoma

b. Basal cell carcinoma

c. Fibrosarcoma

d. Not sure

Which of the following are causative factors for Premalignant disorders and Oral Squamous cell carcinoma?

11. Tobacco use

12. Alcohol consumption

13. UV Light exposure

14. Human Papilloma virus 16 & 18

15. Occupation

16. Genetic

17. Chronic trauma from a sharp tooth

18. Ill-fitting dentures

19. Poor dental hygiene

20. Annual oral cancer examinations should be provided for those of 40 years of age and above

a. Agree

b. Disagree

c. Don’t know

Which of the following are potentially malignant disorders?

21. Oral Submucous Fibrosis

22. Leukoplakia

23. Erythroplakia

24. Lichen Planus

25. Hairy Leukoplakia

26. Smoker's Palate

27. Discoid Lupus Erythematosus

28. Xeroderma Pigmentosum

29. Proliferative Verrucous Leukoplakia

30. Does early detection of OSCC improve the survival status of OSCC?

a. Agree

b. Disagree.

c. Don’t know

31. Are all the OSCCs preceded by PMDs?

a. Agree

b. Disagree

c. Don’t know

32. What is the commonest cause of Oral Submucous Fibrosis?

a. Arecanut/Betel nut

b. Red Chillies

c. Betel quid

d. Don’t know

33. Which of the following PMDs have the highest malignant transformation rate?

a. Erythroplakia

b. Leukoplakia

c. Proliferative verrucous leukoplakia.

d. Don’t know

34. Do patients without habits develop OSCC?

a. Agree

b. Disagree

c. Don’t know

35. Common sites for OSCC in India

a. Tongue and buccal mucosa

b. Palate

c. If other, specify ____________

d. Don’t know

36. Does TNM staging affect the therapeutic modality?

a. Agree

b. Disagree

c. Don’t know

37. Biopsy should be performed of a non-healing ulcer on the tongue?

a. No

b. Yes, if it has a history of 2 weeks

c. Yes, if is present for more than 4 weeks

d. Don’t know

Which of the following are diagnostic aids of OSCC?

38. Toludine blue (Vital Staining)

39. Cytology

40. Brush Biopsy

41. Chemiluminescence

42. Velscope

43. Colposcopy

44. The diagnosis of a clinically suspected case of OSCC is confirmed by

a. Vital staining

b. Cytology

c. Biopsy

d. Don’t know

45. I am comfortable referring suspicious oral lesions to the specialists.

a. Agree

b. Disagree

Thank you for your participation in this survey
